# The role of multisystemic resilience in fostering critical agency: UK adolescents during the COVID-19 Pandemic

**DOI:** 10.1007/s12144-023-04578-1

**Published:** 2023-04-04

**Authors:** Sarah Weidman, Diane T. Levine, Fransiska Louwagie, Kara Blackmore, Linda C. Theron, Dov J. Stekel

**Affiliations:** 1 University of Leicester, Leicester, UK; 2 University of Johannesburg, Johannesburg, South Africa; 3 University of Aberdeen, Aberdeen, UK; 4 University of Pretoria, Pretoria, South Africa; 5 University of Nottingham, Nottingham, UK

**Keywords:** Adolescents, Critical Agency, Critical Consciousness, Resilience, Covid-19

## Abstract

Critical agency (CA) refers to an individual’s feeling of power in relation to social inequalities. Research has demonstrated that high CA is associated with positive adolescent outcomes, however, less is known about what supports are important for its development. Moreover, a large majority of the literature is based on studies from the US and various countries in Africa; although the UK is saturated with inequalities there is little research within a UK context. In this paper we examine (a) the validity of using an existing measure of CA with a sample of UK adolescents and (b) the extent to which resilience supports account for variance in CA. Our analysis identified two distinct factors of CA: justice-oriented and community-oriented. High CA in both factors was explained by resilience supports associated with peer relationships (p < 0.01). Our findings push us towards new relational, ecological ways of understanding adolescent CA. We close by instantiating a translational framework for those devising policies in support of youth resilience and CA.

**Supplementary Information:**

The online version contains supplementary material available at 10.1007/s12144-023-04578-1.

## Introduction

Young people are crucial stakeholders in the contexts of global challenges such as the COVID-19 pandemic and climate change. This is due to both their global demographic, as well as the unique perspectives they can offer to civil society (OECD, 2018). While this has been recognised in some policy spaces such as social welfare (McPherson et al., 2021) and youth justice (Smithson & Jones, 2021), young people’s critical, agentic, and equitable involvement in the design and implementation of programmes intended to address the impacts of risk on their day-to-day lives are currently lacking (Huebner & Arya, 2020; Wong et al., 2021). The involvement of young people is important because both critical agency and resilience are associated with positive outcomes such as well-being, and the kinds of future civic engagement we need to build sustainable futures (Jessee et al., 2021). Consideration of the risks they encounter is equally important because of the subsequent differential impact of risk on outcomes (Ungar & Hadfield, 2019). This study addresses the intersections between these gaps, and proposes key features of the policies and programmes that need to be in place to foster youth critical agency, while accounting for the resilience supports that are needed for young people to survive and thrive.

### Critical agency

Brazilian educator and philosopher Freire defines critical consciousness as “learning to perceive social, political, and economic contradictions, and to take action against the oppressive elements of reality” (Freire, 2000). Consequently, an individual who is critically conscious should understand that certain policies unfairly disadvantage particular groups, and may seek to change such policies. An individual who is less critically conscious, on the other hand, may still be surrounded by such policies, but may not have the means to reflect on these systems as oppressive. The three components of critical consciousness, originally identified by Freire and now operationalised by existing measures (see Diemer et al., 2015), include (a) critical reflection/awareness, (b) critical agency/self-efficacy, and (c) critical action/behaviour. Critical reflection concerns an individual’s understanding of societal inequalities; critical agency—also referred to as political agency or sociopolitical efficacy—concerns an individual’s motivation and feeling of power to create change to the perceived inequalities; and critical action concerns an individual’s actual behaviour in creating change. Researchers have considered critical consciousness to be both a catalyst for, and outcome of, civic engagement (Ajaps & Obiagu, 2020; Thomas et al., 2014).

In this article, we focus specifically on critical agency for a number of reasons. Firstly, research has demonstrated that high critical agency, unlike high critical reflection, is associated with largely positive youth outcomes (Godfrey et al., 2019). To explicate, whereas high critical reflection may be related to positive outcomes (e.g., an understanding of systematic inequalities and therefore an ability to navigate obstacles), it can also be associated with negative outcomes. Specifically, Godfrey et al., (2019) found that when high critical consciousness was paired with low critical agency (e.g., an understanding of systematic inequalities, but a lack of trust in the government or little belief that they can change these structures) young people had notably low scores on measures of socio-emotional and academic well-being. Secondly, unlike critical action, critical agency is not directly dependent on current access to specific opportunities; many of the items on measures of critical action pertain to an individual’s direct participation in political activism events (e.g., “I am involved in activities or groups against racism and discrimination”). In the context of young people in UK schools, this is largely a measure of the school opportunities available to an individual, such as the presence of a student government association. In contrast, critical agency captures the motivation and intention of an individual to take part in such activities, even if in the future (in contrast to critical action, which comprises action actually taken). Finally, critical agency specifically concerns the extent to which an individual trusts the government and feels that they have a role in future decisions. Given research demonstrating that the COVID-19 pandemic had a negative effect on government trust (Defeyter et al., 2021; Williams et al., 2020), and that young people felt excluded from decision making processes (Day et al., 2020), we suggest that critical agency is of particular importance in the current political climate. The following sections will review research that further explores the importance of critical agency during adolescence.

#### Critical agency and positive outcomes in adolescents

Our study focused on critical agency in young people aged 15 to 18 years-old living in the UK. This stage of adolescence is widely understood as important in relation to the development of capacity for critical thinking (Erikson, 1968). Recent research has also demonstrated significant growth in critical consciousness, specifically, during adolescence (Seider et al., 2020). The positive relationship between high critical consciousness and positive outcomes in adolescents has become progressively apparent with research. In an early study by Diemer and Blustein (2006), conducted before the development of standardised measures of critical consciousness, researchers quantified critical consciousness using scales of social dominance orientation (SDO; Pratto et al., 1994) and sociopolitical control (SPCS; Zimmerman & Zahniser, 1991). Findings demonstrated that critical consciousness was positively associated with all measures of career development—including vocational identity, career commitment, and work salience. These results were further supported by Diemer and Hseih (2008), using data from the National Education Longitudinal Study (NELS; Curtin et al., 2002) to explore the association between sociopolitical development and vocational expectations in US adolescents, specifically in students of colour ages 17 to 18-years old identified as coming from a lower SES background. This comprised a very large sample of 1,784 students. Once again, sociopolitical development (a proxy of critical consciousness) accounted for significant variance in vocational expectation. Notably, the items related to family discussion (“how often do you discuss current social and political events with parents or guardians?”) and motivation/agency (“how important is it to help one’s community?”) accounted for this variance.

While these studies demonstrate that high critical consciousness is associated with positive vocational/career development, the measures used did not specifically isolate critical agency from the other aspects of critical consciousness. Helpfully, more recent work has done so. McWhirter and McWhirter (2016) developed and validated a Measure of Adolescent Critical Consciousness (MACC) over two studies, and used it to explore the relationship between critical consciousness and positive vocational development in a sample of Latina/Latino youth. This measure, also used in the current study, separates critical agency from the other components of critical consciousness. In their sample of 476 Latino/Latina high school students, McWhirter and McWhirter reported significant differences in critical agency between students who planned to attend 4-year colleges and those that planned to attend 2-year colleges/ no further education, with students planning to attend 4-year colleges demonstrating higher critical agency. In the second study, with a different sample of 870 students, the researchers reported a significant negative correlation between critical agency and thoughts of dropping out. Critical agency is thus not only associated with positive outcomes in relation to career development, but also academic and socio-emotional well-being (Clonan-Roy et al., 2016; Godfrey et al., 2019). Godfrey et al. (2019) used latent class analysis (LCA) to identify four separate profiles of critical consciousness (e.g., low critical reflection, high critical agency, high critical action versus high critical reflection, low critical agency, low critical action). Individuals who were identified as profile four (high levels of critical reflection and action, but low levels of critical agency) had significantly lower academic confidence, academic engagement, and higher levels of depression than their counterparts with high critical agency. These results are one such motivation for our focus on critical agency.

#### Critical agency in the context of the UK and COVID-19

Researchers have only begun to quantify critical consciousness as its own construct relatively recently (Diemer et al., 2017; McWhirter & McWhirter, 2016; Thomas et al., 2014). Moreover, as evidenced by the studies discussed in the previous section, researchers exploring the importance of critical consciousness have largely done so with marginalised populations within the US. Indeed, in a systematic review of research on adolescent critical consciousness Heberle et al. (2020) reported that out of 67 studies identified, all but three (in Australia, Mexico, and El Salvador) were conducted in the US. On the other hand, to our knowledge, no work exploring critical consciousness during adolescence has been carried out in the UK. We suggest that the focus on critical consciousness in the US is likely due to the widespread literature around the rife racial and socio-economic inequalities in the country. However, the deep structural inequalities in the UK, which fundamentally influence children and young people, have been prevalent long before restrictions associated with COVID-19 were introduced (Thomson et al., 2021).

The UK has also suffered disproportionately high levels of COVID-19 infection and death (Johns Hopkins CRC, 2022). The emergent COVID-19 literature suggests that physical distancing, lockdowns, quarantines, and media rhetoric have led to many already-marginalised young people feeling further alienated (Day et al., 2020). Concern has been expressed about potential long-term implications for individuals and across wider societies in relation to future employment (Costa Dias et al., 2020), interruptions to already problematic national examination systems, possible widening ‘gaps’ in learning (Montacute & Cullinane, 2021), and amplification of inequalities both at home and at school (Andrew et al., 2020). Markedly, young people from low income and/or single-parent households are most likely to experience negative consequences from school closures, particularly access to necessary resources for digital learning (e.g., a computer and appropriate space to study) (OECD, 2020). The complex arrangement of risk factors presented by the pandemic (as well as, for example, Brexit; Warren & Bordoloi, 2020) have had a demonstrable impact on UK’s young people, their families, and their schools (Bayrakdar & Guveli, 2020).

In summary, young people in the UK, particularly from socio-economically disadvantaged backgrounds, are tasked with navigating many social inequalities as they move into adulthood, particularly in the wake of the COVID-19 pandemic. Critical agency is often pivotal in relation to ensuring positive outcomes, as are the supports underpinning resilience which are needed to survive and thrive in conditions of chronic or intermittent challenge.

### Multisystemic model of resilience and critical agency in adolescents

Recent years have seen a re-framing of understandings of resilience in adolescence. Discourses and definitions have progressed from largely focusing on internal and individual traits or characteristics (Kumpfer, 1999), through to more socio-ecological framings including considerations of family (Walsh, 2002) or educative environment (e.g. Masten et al., 2021). The socio-ecological approach and emergent related theory has generated helpful questions for scholars and practitioners aiming to establish which interventions might be most impactful for which young people, and under which circumstances (Ungar, 2018).

Contemporary scholarship now largely agrees that resilience of any individual adolescent is dependent on the successful actions and interactions in multiple surrounding systems, recently termed the ‘multisystemic’ approach (Clark, 2022; Luthar et al., 2021; Masten et al., 2021; Masten & Motti-Stefanidi, 2020; Matsopoulous & Luthar, 2020; Ungar & Theron, 2020). Ungar and Theron demonstrate that resilience is best understood as a set of processes in which protective and promotive factors and processes (PPFPs) (both internal and external) interplay to support individuals’ ability to adapt to adversity (2020).

The relationship between resilience pathways and critical agency is the focus of the current paper. Specifically, we aim to better understand what resources are necessary in order for students to develop a sense of power that they have the ability to make a difference in relation to existing inequalities they experience or witness, and for that sense of power to help them cope in adversity. Like multisystemic resilience, critical agency can be considered dynamic in that it develops with both age and experience. Factors which influence growth in critical agency include not only demographic variables (e.g., gender), but also presence of educational programmes (Seider et al., 2019) and teaching practices (Jagers et al., 2017). We agree with Godfrey and Burson (2018) who suggest that research on critical consciousness should focus on ‘marginalising systems’ rather than ‘marginalised individuals,’ and argue for a need to concentrate on the many interacting factors which create such systems. In relation to the multisystemic model of resilience proposed by Ungar and Theron (2020), the studies discussed demonstrate how the development of critical agency is influenced by the interaction of biological and psychological systems in addition to social and built environments (as indicated by the significant associations between related constructs and gender, family, school, and community relationships).

These studies do not, however, explain the interrelationships between critical agency and the systems needed to adapt when life presents challenges, that is, the processes and practices of resilience. In the current study, we are specifically concerned with the association between these different systems of resilience and critical agency. In doing so, we build on the work of Clonan-Roy et al. (2016) in which they propose a critically feminist model that centres critical consciousness, resistance, and resilience in understanding the development of girls of colour. Their work, as ours, aligns with the Positive Youth Development (PYD) model (Lerner et al., 2009)—which suggests that thriving is associated with making positive contributions to society. We hypothesise that the presence of self-reported resilience resources should predict high critical agency scores.

## Research questions and hypotheses

In this study, we aimed to better understand the relationship between resilience supports and critical agency. Our research questions and hypotheses are presented below.

- *What is the factor structure of the Critical Agency scale of the Measure of Adolescent Critical Consciousness (MACC), within a sample of UK adolescents?*

An overwhelming majority of the research on critical consciousness has been conducted with adolescents in the US (Heberle et al., 2020). Accordingly, no recorded measures of critical agency have been used with samples of adolescents in the UK. Before exploring the association between critical agency and multisystemic resilience we first examined the feasibility of using the MACC within our sample. As discussed in the previous section, the measure used in the present study was originally validated in a large sample of Latino/Latina students from 65 high schools across the US. From an initial ten items, researchers used a factor analysis to identify two factors: critical agency (7 items) and critical behaviour (3 items). For the purposes of our study, we use the seven identified items to quantify students’ critical agency. Given the markedly different sample of UK respondents in our study, we run an exploratory factor analysis to understand if these items load onto a single factor or multiple factors. We hypothesise that the measure will demonstrate reasonable inter-item reliability in our sample of UK young people.

(b) *Do resilience resources significantly predict variance in critical agency? Which specific resilience pathways are associated with high critical agency?*

In line with previous research, we hypothesise that individual differences in critical agency will be accounted for by resilience supports. We acknowledge that the complexity of this landscape mitigates against a universal or ‘complete’ understanding, but aim to provide novel insight into the interplay between these two commonly-used constructs.

## Method

### Context of data collection

Data for this study was collected as part of an Arts and Humanities Research Council project (AH/V015060/1) Covid in Cartoons project, conducted by researchers from the University of Leicester in partnership with Shout Out UK and Cartooning for Peace. The project aimed to educate young people about the medium of political cartoons and to encourage them to use this medium to make meaning of their own experiences of the COVID-19 pandemic. As part of this course, students completed questionnaires about their own experience of the pandemic, in addition to measures of resilience pathways and critical agency. The latter two survey measures are used for analysis in the current article. All surveys were completed between September 2021 and March 2022.

### Participants

Participants included 370 students, ages 15 to 18-years-old (mean age = 16.5, *SD* = 0.9; approximate as calculated from class year), from 16 schools/organisations across the United Kingdom. Schools were recruited to take part in the Covid in Cartoons project through a partnership with Shout Out UK (SOUK), a youth political literacy platform. SOUK reached out to schools across the UK, largely prioritising those schools with a high percentage of young people registered as benefiting from Free School Meals. Schools were chosen based on interest in the mini-course from the administration and class teachers. Mini-course and surveys were delivered to all classes who took part. Ethical approval for this project was granted by University of Leicester (Ethics application 28,828). Students, teachers, and parents at participating schools were provided with information sheets and consent forms outlining the research goals and curriculum before taking part in the study. Out of the 370 students, 226 identified as male (61.1%), 130 as female (35.1%), 7 as non-binary (1.9%) and 7 preferred not to share their gender (1.9%). Ethnicity of participants was moderately diverse, with 40.8% of students identifying as White, 37.3% of students identifying as Asian/ Asian British, 10.0% of students identifying as Black/ African/ Caribbean or Black British, 2.7% identifying as Mixed, and 9.2% identifying as another ethnicity. For more information about the demographics of the schools, see Table 1.

**Table 1:** Demographics of Participating Schools

School ID	Number Surveys	School Info	
		School Type	% Eligible Free Meal
A	74 (20.0%)	Independent	0%
B	46 (12.4%)	Academy	24.0%
C	26 (7.0%)	College	*NR*
D	39 (10.5%)	Community	27.6%
E	11 (3.0%)	Community	18.4%
F	62 (16.8%)	Community	21.9%
G	18 (4.9%)	Community	31.0%
H	7 (1.9%)	Academy	24.7%
I	5 (1.4%)	Foundation	*NA*
J	16 (4.3%)	Community	30.4%
K	10 (2.7%)	College	*NR*
L	9 (2.4%)	Community	10.4%
M	9 (2.4%)	Academy	41.1%
N	22 (5.9%)	Academy	31.2%
O	10 (2.7%)	Independent	0%
P	6 (1.6%)	Academy	41.20%

School types in the UK: The UK government website specifies that Academy schools are funded by the government and run by an ‘academy trust’. While not fee-charging, they have more control over their term times and curriculum than Community schools. They are inspected regularly and follow national rules on admissions, special educational needs, and exclusions as other state-funded schools. Colleges are places students can study after age 16 – they take different forms such as sixth form colleges or further education colleges (largely vocational). Community schools (also known as ‘local authority-maintained schools’) are state-funded schools that are not influenced by business or religious groups. They follow the national curriculum, are inspected regularly, and follow all national rules. Foundation schools are funded by government via local authorities, but have a little more freedom than community schools. Foundation schools can be supported by religious groups. Independent schools (also known as ‘private’ schools) charge fees to attend, and pupils are not required to follow the national curriculum. They are not funded by the government, but are registered and inspected regularly (Schools and education, n.d.)

*NR*: Not Reported, *NA*: Not Applicable.

### Survey

Surveys were completed either in paper during class, or on-line via the survey platform Qualtrics (during class or at home), depending on the teacher’s schedule. Surveys (detailed below) included a demographic questionnaire (gender, ethnicity), a measure of resilience, and a measure of critical agency, in addition to questions about the students’ experience of the pandemic, their knowledge of political cartoons, and their attitudes towards humour. For the purpose of this paper, only measures of resilience and critical agency will be included in analysis. Analysis of the data was carried out using both IBM SPSS (version 26) and R (version 4.2.2).

#### Critical agency (Measure of adolescent critical consciousness)

As discussed earlier, critical agency was quantified using the critical agency items of Measure of Adolescent Critical Consciousness (MACC; McWhirter & McWhirter, 2016). This measure was selected for use in the current study for two reasons. Firstly, unlike other existing measures of critical consciousness, this measure specifically quantifies the critical agency component of critical consciousness. Secondly, the measure was originally designed for use in a sample of adolescent participants in another ‘Global North’ country (USA) (though we acknowledge this term can be problematic). However, unlike our sample, it is notable that the sample of adolescents in which this measure was originally validated were Latina/Latino individuals. Accordingly, the authors explained that their “focus was on racism and discrimination, as we expected this to be the most salient aspect of inequity experienced by our Latina/Latino adolescent participants” (McWhirter & McWhirter, 2016, pg. 545). In our sample, we also expected racism and discrimination to be a prominent concern. However, given the more diverse sample of our participants and the different context, we specifically assessed whether the items on this scale (a) loaded onto a single factor and (b) demonstrated reasonable inter-item reliability. This will be addressed in the following section. This measure includes 7-items that participants rate on a 4-point scale (Strongly Disagree, Disagree, Agree, Strongly Agree). All items are in Table 2.

**Table 2:** Critical Agency Items

Critical Agency Measure: MACC Items
(1) There are ways that I can contribute to my community
(2) I am motivated to try to end racism and discrimination
(3) It is important to fight against social and economic inequality
(4) I can make a difference in my community
(5) More effort is needed to end racism and discrimination
(6) It is important to me to contribute to my community
(7) In the future, I will participate in activities or groups that struggle against racism and discrimination

#### Resilience (CYRM-R)

The 5-point version of the Child & Youth Resilience Measure (CYRM-R; Jefferies et al., 2019) was used to quantify resilience pathways. In the Youth form of this measure, designed for use with 10 to 23-year-olds, participants respond to items concerning their own perceived resilience resources (e.g., “My friends care about me when times are hard”) on a 5-point scale (Not at all, A little, Somewhat, Quite a bit, A lot). 10-items from the full 17-item scale were used in this study. These items were originally selected in order to be sensitive to safeguarding issues surrounding the anonymous administration of the measure in a school setting (i.e., items which may require follow up, such as ‘I feel safe when I am with my family/caregiver(s)’ or ‘If I am hungry, there is enough to eat’ were removed from the measure). The remaining items were then selected to be consistent with the validated sub-scale of personal resilience (Jefferies et al., 2019). A list of all items can be found in Table 3. This measure demonstrated excellent internal reliability in our sample, with Cronbach's alpha for this measure (α = 0.88).

**Table 3:** Resilience Measure Items

Resilience Measure: CYRM-R Items
(1) I get along with people around me
(2) Getting an education is important to me
(3) I know how to behave/act in different situations (such as school, home and church)
(4) People like to spend time with me
(5) I feel supported by my friends
(6) My family/caregiver(s) care about me when times are hard (for example if I am sick or have done something wrong)
(7) My friends care about me when times are hard (for example if I am sick or have done something wrong)
(8) I am treated fairly in my community
(9) I have chances to show others that I am growing up and can do things by myself
(10) I have chances to learn things that will be useful when I am older (like cooking, working, and helping others)

## Results

### Research question one: Factor structure of MACC (Critical agency)

Our first research question concerned the feasibility of using the MACC to quantify critical agency in our sample of young people in the UK. In order to address this aim, scores on Critical Agency items were considered. Overall, scores on the critical agency were spread across the full possible range of the measure, with scores between 7 (all lowest possible responses) and 28 (all highest possible responses). Notably, scores tended to be on the higher end, with a mean of 22.4. Bartlett’s test of sphericity, 1245.697, *p* < 0.001 demonstrated a high correlation between items of the MACC. Therefore, a factor analysis using Principal Axis Factor (PAF) with Varimax (orthogonal) rotation was conducted. An examination of the Kaiser-Meyer Olkin measure of sampling adequacy suggested that the sample was factorable (KMO = 0.868). The results yielded that all seven items loaded onto two factors, containing 72.0% of variance in the data. See supplementary materials for table of eigenvalues and comparison of this two-factor model with a (poorer fit) single factor model.

On examination of the items that loaded onto these two factors, two themes were identified: Factor 1 contained items that specifically concerned justice around racism and discrimination (eigenvalue of 4.02, accounting for 57.46% of the variance), while Factor 2 contained items that concerned contribution to the community (eigenvalue of 1.02, accounting for additional 14.57% of variance) (Table 4). These factors were moderately inter-correlated (0.63) and both exhibited good internal consistency and reliability: Justice-Oriented Critical Agency (Cronbach’s alpha α = 0.80), Community-Oriented Critical Agency (Cronbach’s alpha α = 0.86).

**Table 4:** Factor Loadings and Communalities for Principal Axis Factor (PAF) with Varimax (orthogonal) for Critical Agency Items

Item	Factor 1 (Justice-Oriented)	Factor 2 (Community- Oriented)	Communalities
There are ways that I can contribute to my community	(0.187)	**0.627**	0.376
I am motivated to try to end racism and discrimination	**0.780**	(0.304)	0.587
It is important to fight against social and economic inequality	**0.696**	(0.384)	0.552
I can make a difference in my community	(0.262)	**0.817**	0.519
More effort is needed to end racism and discrimination	**0.704**	(0.133)	0.426
It is important to me to contribute to my community	(0.504)	**0.618**	0.570
In the future, I will participate in activities or groups that struggle against racism and discrimination	**0.687**	(0.372)	0.562

### Research question two: Association between critical agency and resilience

Our second research question concerned the relationship between critical agency and resilience pathways in our sample of UK young people. Firstly, descriptive statistics for all measures (including the two new factors of Critical Agency) were examined (Table 5). All data was distributed approximately normally, with appropriate values of skewness and kurtosis (± 3).

**Table 5:** Descriptive Statistics for all Constructs

Construct	Min	Max	Mean	SD	Skewness	Kurtosis
Critical Agency	7.0	28.0	22.4	3.8	-1.0	2.3
Factor 1 (Justice)	4.0	19.0	13.3	2.4	-1.2	2.2
Factor 2 (Community)	3.0	12.0	9.0	1.8	-0.6	1.2
Resilience	10.0	50.0	40.0	7.0	-1.1	2.5

Bivariate correlations were run to explore the relationship between both factors of critical agency, overall resilience, and reported demographics. The results demonstrated that resilience was moderately positively associated with both justice-oriented Critical Agency (*r* = 0.393, p < 0.001) and community-oriented Critical Agency (*r* = 0.446, p < 0.001).

Given previous research demonstrating associations between both critical consciousness and resilience with demographics such as gender (McWhirter & McWhirter, 2016), ethnicity (Godfrey et al., 2019), and socio-economic status (Roy et al., 2019), further analyses were run to explore whether these measures were associated in the current study. The results demonstrated that, in this study, score on the resilience measure was not associated with what might loosely be described as ‘community resources’ (defined by percentage of students receiving free school meals at school), participant gender, or participant ethnicity (Table 6). However, being male was negatively associated with both justice-oriented and community-oriented Critical Agency (*p* < 0.001). Also, there was a small positive association between participants who reported being of a mixed ethnicity background and high scores on the community-oriented Critical Agency factor, however, this did not survive a Bonferroni correction (*p* = 0.15).

**Table 6:** Correlations between Critical Agency, Resilience, and Demographics *(N* = *370)*

Construct	Community Resources^a^	Gender (Male)	White	Asian	Black	Mixed	Other
Critical Agency (Justice)	0.01	-0.31**	-0.05	0.06	-0.07	0.09	-0.01
Critical Agency (Community)	0.01	-0.17**	-0.05	0.05	-0.08	0.14*	0.02
Resilience	0.03	0.04	-0.01	0.08	-0.10	-0.01	-0.01

^**a**^1 = More than 20% FSM, 2 = Less than 20% FSM, **p < 0.001, *p < 0.01

Regression analysis was used to explore whether resilience items contributed unique variance to both factors of critical agency. Prior to conducting the analysis, the variance inflation factor (VIF) values were calculated to check for multicollinearity. All values were below 3, indicating no strong correlations between the resilience items. Given that gender was significantly associated with both factors of CA, this was entered into step one of a hierarchical multiple linear regression. Results demonstrated that, at step one, gender contributed significantly to the model predicting justice-oriented critical agency *F*(1,368) = 39.35, *p* < 0.001, and accounted for 10% of variance. At step two, resilience pathways accounted for an additional 19% of unique variance, and this change in R^2^ was significant *F*(11,358) = 12.82, *p* < 0.001. Specifically, two items accounted for all unique variance: ‘*My friends care about me when times are hard’* and *‘I have chances to show others that I am growing up and can do things by myself’* (Table 7).

**Table 7:** Resilience Pathways predicting Critical Agency (Justice) (*n* = *370)*

Critical Agency (Justice)	R	R^2^	ΔR^2^	β	*t*	B	*p*
Step 1	0.31	0.10	0.10				
Gender (Male)				**-0.31**	**-6.3**	**-1.5**	**0.00**
Step 2	0.53	0.29	0.19				
Gender (Male)				**-0.33**	**-6.85**	**-1.61**	**0.00**
I get along with people around me				0.04	0.71	0.09	0.48
Getting an education is important to me				-0.03	-0.45	-0.07	0.65
I know how to behave/act in different situations				0.10	1.69	0.23	0.09
People like to spend time with me				0.01	0.14	0.02	0.89
I feel supported by my friends				0.10	1.86	0.24	0.07
I feel that I belong at my school				0.09	1.39	0.22	0.17
My friends care about me when times are hard				**0.13**	**2.21**	**0.34**	**0.03**
I am treated fairly in my community				0.02	0.36	0.05	0.72
I have chances to show others that I am growing up (…)				**0.17**	**2.54**	**0.38**	**0.01**
I have chances to learn things that will be useful (…)				-0.01	-0.21	-0.04	0.84

Significant items in bold

Results also demonstrated that, at step one, gender contributed significantly to the model predicting community-oriented critical agency *F*(1,368) = 10.34, *p* = 0.001 and accounted for 3% of variance. At step two, resilience pathways accounted for an additional 24% of unique variance, and this change in R^2^ was significant *F*(11,358) = 11.83, *p* < 0.001. Specifically, three items accounted for all unique variance: ‘*I feel supported by my friends,” “My friends care about me when times are hard’* and *‘I have chances to show others that I am growing up and can do things by myself’* (Table 8).

**Table 8:** Resilience Pathways predicting Critical Agency (Community) (n = 370)

**Critical Agency (Community)**	R	R^2^	ΔR^2^	β	*t*	B	*p*
Step 1	0.17	0.03	0.03				
Gender (Male)				**-0.17**	**-3.22**	**-0.61**	**0.00**
Step 2	0.52	0.27	0.24				
Gender (Male)				**-0.20**	**-4.06**	**-0.71**	**0.00**
I get along with people around me				0.10	1.65	0.16	0.10
Getting an education is important to me				0.01	0.83	0.01	0.93
I know how to behave/act in different situations				0.08	1.35	0.14	0.18
People like to spend time with me				-0.02	-0.24	-0.03	0.81
I feel supported by my friends				**0.24**	**4.24**	**0.41**	**0.00**
I feel that I belong at my school				0.02	0.35	0.04	0.72
My friends care about me when times are hard				**0.18**	**2.39**	**0.28**	**0.02**
I am treated fairly in my community				**0.02**	0.37	0.04	0.71
I have chances to show others that I am growing up (…)				**0.13**	**1.20**	**0.23**	**0.04**
I have chances to learn things that will be useful (…)				-0.02	0.30	-0.04	0.77

Significant items in bold

## Discussion

Our findings suggest new insights into the nature of critical agency in a sample of diverse adolescents in England, and an observed interplay between resilience resources and the development of critical agency for this sample during a period of high adversity. Here, we focus on three interrelated findings emerging from the data. First, we consider critical agency within our specific sample and implications for future research. Second, we highlight the importance of fostering supportive peer relationships and providing opportunities to demonstrate growth for adolescents, potentially encouraging sociopolitical efficacy. Finally, results allow us to begin to conceptualise the relationship between critical agency and resilience within a UK pandemic context. We offer this as a framework that describes a set of *potential* risk, protective, and promotive factors leading to positive adaptive outcomes.

This study confirmed the feasibility of using the Measure of Adolescent Critical Consciousness within a sample of English adolescents. The two identified factors speak to the dual structure of the measure, suggesting that future research consider the justice-oriented and community-oriented facets as potentially separate constructs. Upon review, the items identified as ‘justice-oriented’ (2, 3, 5 and 7) all explicitly concerned ‘racism, discrimination, or inequality’ whereas the items identified as ‘community-oriented’ (1, 4, and 6) all explicitly referred to the ‘importance of’ or ‘contribution to’ community.’ We suggest that the duality of the measure in our sample is potentially due to the diversity of students; the sample of young people in the current study were considerably more diverse in ethnicity and resources than the original sample (of all Latino/ Latina participants) in which the measure was validated (McWhirter & McWhirter, 2016). To elucidate, while all young people who took part in this study may have been exposed to the systematic inequalities associated with living in the UK during COVID-19, factors such as gender, ethnicity and family/ community resources may mediate direct exposure to discrimination, racism, and inequality. This is reflected in research exhibiting an association between critical consciousness and both exposure to violence and income inequality (Roy et al., 2019) and research demonstrating higher levels of critical consciousness in youth of colour, who have likely experienced more discrimination or racism, than White youth (Diemer & Li, 2011). Accordingly, we suggest justice-oriented, rather than community-oriented, critical agency *may* be more closely related to these factors and suggest this may be an area ripe for future research.

Although neither ethnicity nor ‘community resources’ (a limited proxy indicated by percentage of free school meals for this particular study) were significantly associated with either factor of critical agency in this study, we suggest this is likely because the current sample was not large or balanced enough (note the spread of student ethnicity) to demonstrate such patterns. Similarly, it may be that the proxy was not sufficient to capture the richness of ‘community resources’. Nevertheless, we argue that, in accordance with research demonstrating associations between ethnicity and patterns of critical consciousness (Godfrey et al., 2019), the importance of ethnicity and community resources in relation to critical agency should not be understated and requires further investigation.

In contrast, the significant association between gender and critical agency—particularly justice-oriented critical agency—is of note: participants who identified as male scored significantly lower on these measures. This effect of gender was also reported by McWhirter and McWhirter (2016), with female participants scoring higher on the MACC than male participants. The authors suggest that this may be due to the higher likelihood of Latina girls’ experience of sexism, which may lead to greater motivation for combating inequality. This claim is also echoed by Diemer et al. (2006) who found that female participants voiced stronger motivation for taking action against sexism than male participants. Our results suggest that this may also be the case in our sample of UK adolescents who identify as female or non-binary. Although the data does not allow us to explore this further, we suggest that future research consider the role of gender, and potential sexism, in relation to critical reflection and agency.

This study also highlights the significance of friendship and socialisation in relation to critical agency. The importance of peer support for adolescent development is already well established; in an integrative review of 15 fifteen studies exploring the relationship between peer support and mental health, Roach (2018) found that higher levels of prosocial peer support was consistently associated with less depressive symptoms and better stress management. However, the potential role of peer support in relation to critical agency—both motivation to work towards justice and contribute to one’s community—is less well documented. Studies that have explored the importance of socialisation in relation to critical consciousness have largely focused on the importance of family, instead of, or in combination with, peer support (Heberle et al., 2020). In the above-mentioned study by Diemer and Li (2011), researchers found that critical agency was positively associated with parental and peer support— operationalised by how often students talked about ‘current events or things that you heard in the news with your family and friends.’ This study suggests that the role of peer support may be mediated by the discussion of politics, specifically, rather than general discussion. However, other research has demonstrated that simply interacting with peers– for example, learning to play chess at a community centre (Fegley et al., 2006) or taking part in an LGBTQQ theatre group (Wernick et al., 2014) can lead to an increase in critical reflection. Scholars have also suggested that socialisation may be important in itself, simply by allowing others to hear perspectives of their peers (Clark & Seider, 2017).

Our study is also in line with research exploring the likelihood of young people’s involvement in civic engagement. Jagers et al. (2017) reported that in a study with 515 Black and Latino 11 to 14-year-olds, civic engagement behaviours, attitudes, and beliefs (roughly aligned with critical action, agency, and reflection) were predicted by positive ‘homeroom practices’—such as including the opportunity to build community with other students. Moreover, higher scores on homeroom practices also longitudinally predicted civic engagement attitudes (most closely aligned with critical agency) but not behaviour or beliefs. The researchers suggest that these results speak to the importance of allowing young people to have social interactions which help them learn about themselves and develop competencies that support civic engagement and well-being. We suggest that the results of the current study unreservedly support this argument.

Of course, one of the differences between Jagers et al. (2017) and the present study is that our research is particularly concerned with the relationship between critical agency and peer relationships during the COVID-19 pandemic: a markedly difficult time to engage in social interactions. Wray-Lake et al. (2022) explored how adolescents spent their time during the pandemic, measuring interpersonal support including strength of peer relationships, strength of parent relationships, and presence of parental conflict, in addition to the degree to which students chose to help others and engage in political action. In accordance with our results, which show that peer support items made a significant contribution towards variance in community-oriented critical agency, the researchers reported that friendship support was positively associated with helping others (r = 0.12, *p* < 0.001).

Our data suggest a complex but important interplay between critical agency and resilience supports for adolescents living in a time of high adversity. The findings invite us to reflect on a possible fresh direction for understanding critical agency mirroring that of resilience science in recent years, in which we might move distinctively on from the reductive approach of exploring individual ‘traits’ or ‘skills’, towards a much more relational and ecological way of reflecting on the ways in which critical agency manifests in adolescence. In investigating critical agency as a construct in itself in relation to resilience, we inevitably investigate the relational nexuses within which relationships evolve. We have demonstrated that – during this COVID-19 period—youth critical agency has occured within the group discussion that is critical consciousness; our data invites reflection on whether that group discussion inevitably speaks beyond itself.

Crucially, as stated at the outset, our goal has been to propose potential features of youth-focused policies and programmes that foster positive outcomes for critical agency and resilience pathways. In order to do so, we build on the translational framework proposed by Ungar and Theron (2020), in which the authors suggest that there are direct relationships between risk exposure (for example the impact of health protection measures during the pandemic), internal and external protective and promotive factors and processes (PPFP) systems, contextual considerations, and desired outcomes. We began by instantiating our key findings with each of these categories (Table 9).

**Table 9:** Instantiating translational framework (Ungar & Theron, 2020) with dominant findings

Framework dimension	Instantiation of key findings from our data
Risk exposure	Experience of racism and/or discrimination
	Witnessing racism and/or discrimination
	Community-based challenges
Internal PPFP systems	Developmental change
	Motivation to contribute to addressing inequalities
	Intersectionality (stronger findings for gender, suggestion of need for further research focused on ethnicity)
	Sense of belonging to—and commitment to—community
External PPFP systems	Strong peer interaction and support systems
	Opportunities to demonstrate growth and development
	Opportunities to participate in activities intended to address inequalities
	Opportunities to contribute to community and ‘make a difference’
Considerations	Social constructions of gender and adolescence
	Availability of social and economic resources
Desired outcomes	Active civic engagement for publics and personal good
	Criticality of media and other forms of consumption
	Supportive peer group and interaction systems, and respect for these from other elements of the multisystem
	Agentic feelings and practices in relation to developing and supporting community

**Fig. 1:**
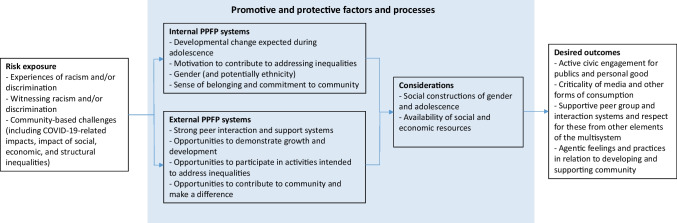
Significant interplays linking critical agency and resilience pathways. Populating Ungar and Theron (2020)

We can then use this instantiation to propose a conceptual framework that might offer some guidance for those designing programmes and policies with and for young people, and those co-producing or delivering research with this constituent group (Fig. 1).

Figure 1 demonstrates the range of exposure to risk reported by our participants during the data gathering period. The focus on racism and discrimination is, of course, dictated by the nature of the survey instrument, but we argue could stand as a proxy indicator for these and other forms of structural or socio-cultural inequalities. Exposure to risk potentiates subsequent adversity, and it is in this context that we then reflect on the internal and external PPFP systems relating specifically to justice- and community-oriented critical agency. We note in particular the centrality of peer interactions and peer support systems for our participants; while this is unsurprising for this developmental age and stage, it invites reflection for both research and practitioners relating to the ways in which we can best understand, articulate, and support healthy interactions between young people.

Building on Heberle et al. (2020) we offer three hypotheses from these reflections, ripe for future research. First, that critical agency could be protective for resilience, particularly in systems where critical agency (and consciousness more widely) is also high. Second, we hypothesise that critical agency could be even more important in systems where chronic structural inequality has led to circumstances where such agency is not understood or valued. And finally, as our data relating to gender suggest, we are concerned that critical agency might take harmful forms for resilience in systems where contexts are maladaptive or represent higher risk (Godfrey et al., 2019). Given these risks, we recommend that those developing policies and programmes aimed at young people consider whether the outcomes we propose in Fig. 1 are indeed desired, and what meaningful actions might be needed to generate those outcomes in light of the relationship we have found between critical agency and resilience.

This is why considerations relating to social constructions of intersectional adolescence are so fundamental; it is difficult to see how peer relationships, attachments and friendships can be addressed respectfully and sustainably without acknowledging (and arguably challenging) the problematic narratives surrounding adolescence in contemporary discourse (Altikulaç et al., 2019). We suggest, however, that the goal is worthy; located in the wider evidence, our data suggest that young people who have critical agentic feelings and practices in relation to themselves, each other, and a wider community of belonging, have both active resilience pathways and better outcomes for positive civic engagement—particularly in the context of the COVID-19 pandemic.

## Limitations

The difficulty of delivering research with young people during the height of COVID-19 restrictions in the UK have been articulated elsewhere (e.g. Salam et al., 2022), and this study was certainly not immune to these challenges. Our primary limitation relates to the short time window at our disposal for gathering data. During a 7-month period coinciding with school teaching terms (September 2021 through March of 2022) we delivered pre- and post-surveys in combination with a four-module minicourse, for which we collected workbooks and carried out focus groups using both virtual and face-to-face methods across participating schools (note that virtual methods were used during lockdown or restricted access periods). During these months, the UK was experiencing an increase in the Delta and Omicron variants of COVID-19 and responding with ‘Plan B’—including measures such as compulsory face masks and use of the NHS COVID-19 app in many indoor venues (Institute for Government, 2022). The project delivery and data collection thus took place at a time of ongoing constraints.

Due to the range in delivery methods and the associated challenges when building and sustaining our sample—including limitations around collecting post-surveys, largely due to the changing and growing pressures placed on schools during this time-period—the analysis is based on the substantial sample of surveys completed before the course (rather than both before and after the course). In this way, our analysis provides understanding of the many connected concepts discussed thus far, but cannot provide longitudinal insight or speak to an evaluation of the associated mini-course. Additionally, scores on the resilience measure can only be ascribed to the reported feelings of these participants, during the process of completing the survey. We therefore advise caution in making over-strong claims during a period of high adversity, and in the absence of longitudinal data. However, it should be noted that some limitations have been addressed through the qualitative work completed during the study which is out of scope for this article and reported elsewhere (Louwagie et al., in preparation).

Furthermore, although our results speak to the development of critical agency of English secondary school students in our 16 participating schools, it is important to note that the diversity of our sample means that many factors (portrayed in the multisystemic model of resilience) that influence adolescents’ feelings of power in relation to social inequalities are absent (for example quality of natural environment/access to green space). Similarly, as a result of the need for pragmatic sampling of schools during the pandemic, more males participated in the study than females. Students in our sample came from 16 schools from communities with considerably different backgrounds and resources. It is also notable that the directional relationship between critical agency and resilience pathways (including peer relationships and opportunity) cannot be determined from our concurrent data. For example, there is a potential for a bidirectional relationship in which students who have developed a higher sense of critical agency are also more likely to seek out close friendship support and seek opportunities for growth. Consequently, we suggest that our results should be viewed as a window into the interplay of relationships between these factors.

## Conclusion

Taken as a whole our results, in partnership with previous work, suggest that fostering strong peer relationships and providing opportunities for growth in educational settings is crucial. We also suggest that while the Critical Agency scale of the MACC is an appropriate measure for use in a diverse UK sample, future studies should consider community-oriented and justice-oriented factors as potentially separate but interrelated factors. Notably, research on motivation and involvement in creating social change suggests that engagement with these processes in adolescents predicts engagement in adulthood citizenship and volunteering in adulthood (Eckstein et al., 2013; Obradović & Masten, 2007)— suggesting that the fostering of critical agency during adolescence has lasting effects. According to Watts et al., (2011), “if there is a single term that captures [critical consciousness] practice, it is group discussion” (pg., 54). This is largely because the development of critical consciousness is social in nature; critical reflection involves an understanding of others’ experiences and critical agency involves taking part in a collective action to make a change. Particularly given studies demonstrating a growth in critical consciousness in the presence of youth civic development opportunities (e.g., school-based civic programming and opportunities to engage with activism) (Jagers et al., 2017), we suggest future work documenting the effects of these programmes and their interplay with resilience is key. The inclusion or programmes to foster peer support as resilience pathways should be prioritised, with the development of critical agency as one of the many anticipated positive outcomes.

## Supplementary information

Below is the link to the electronic supplementary material.

Supplementary file1 (DOCX 15.2 KB)

## Data Availability

The datasets generated during and/or analysed during the current study are available in the University of Leicester Figshare repository: 10.25392/leicester.data.c.6079419.v2..
