# Supplementary material for: The role of multisystemic resilience in fostering critical agency: UK adolescents during the COVID-19 Pandemic
Source: Curr Psychol. 2023 Apr 4:1–15. Online ahead of print. doi: 10.1007/s12144-023-04578-1 (PMC10072812; doi:10.1007/s12144-023-04578-1)
Supplement: Supplementary file 1 — Supplementary file1 (DOCX 15.2 KB) [file 12144_2023_4578_MOESM1_ESM.docx]

**Supplementary Materials:**

**Table A.** Eigenvalues, Percentages, and Cumulative Percentages for Critical Agency Items

| **Factor** | **Eigenvalue** | **% of Variance** | **Cumulative** |
| --- | --- | --- | --- |
| 1 (Justice-Oriented) | 4.02 | 57.46 | 57.46 |
| 2 (Community-Oriented) | 1.02 | 14.57 | 72.03 |

**Table B.** Comparison of CFA results for a single-factor and two-factor model

| **Model** | **df** | **χ2** | **SRMR** | **RMSEA** | **AIC** | **BIC** | **CFI** | **TLI** |
| --- | --- | --- | --- | --- | --- | --- | --- | --- |
| Single-factor | 14 | 150.66 | 0.07 | 0.162 | 4505 | 4560 | 0.89 | 0.84 |
| Two-factor | 13 | 73.703 | 0.05 | 0.112 | 4430 | 4489 | 0.95 | 0.92 |

Note. The two-factor model of the MACC items demonstrates a better fit than the single-factor model
